# Supplementary material for: Autonomous medical evaluation for guideline adherence of large language models
Source: NPJ Digit Med. 2024 Dec 12;7:358. doi: 10.1038/s41746-024-01356-6 (PMC11638254; doi:10.1038/s41746-024-01356-6)
Supplement: Supplementary file 1 — Supplementary Information [file 41746_2024_1356_MOESM1_ESM.pdf]

Supplementary Data 1: Details on the LLM prompts and on the evaluation algorithm

Details on the LLM prompts

Candidate Model:
"Initial Case: {*case_text*}
Question: {*question*}"

Evaluator Model:
"Evaluate the text below given the criteria list. Therefore, return a list of True or False for each criterion, depending on whether the text below meets this criterion or not. Do not evaluate each bullet point of the text separately. Do not justify your decision.
Text: {*candidate_response*}
Criteria: {*criteria_list*}"
By employing an LLM-as-a-judge technique, similar to the one validated in the AMIE (Articulate Medical Intelligence Explorer) evaluation framework, GPT-4 can effectively assess factual accuracy and guideline adherence. It is particularly suitable for evaluating complex medical decision-making tasks, where traditional evaluation metrics fall short, as it goes beyond simple factual recall and requires deeper understanding and reasoning.

Details on the evaluation algorithm

Given *Candidate Model's* response $c^{(i)}$ to the i-th question, evaluation criteria $K^{(i)}$ and amount of evaluation attempts n, the recursive function $evaluate(c^{(i)}, K^{(i)}, n)$ can be defined as follows.

1. Set *fail rate* of the run $\lambda = 1$ to initiate the while-loop
2. While $\lambda > 0.5$:
   a. Let $E = \{e_1, e_2, \ldots, e_n\}$ be the *Evaluator Model's* outputs of n parallel evaluations of $c^{(i)}$ based on criteria $K^{(i)}$ with l being the amount of criteria.
   b. For each evaluation output e_j:
      i. Extract list of boolean b_j
      ii. Check validity of the attempt j: $v_j = 1\ if\ |b_j| = l, 0\ otherwise$
   c. Count number of valid evaluations: $\hat{n} = \sum_{j=1}^{n} \quad v_k$
   d. Calculate the *fail rate* of the evaluation run: $\lambda = 1 - \frac{\hat{n}}{n}$
   e. If $\lambda \leq 0.5$:

i. For every criterion, calculate the mean score based on the valid evaluation attempts and the final result using majority vote:

$$r_k = \sum_{j=1}^{\hat{n}} \frac{v_k}{\hat{n}}$$

$$Maj_k = \lfloor r_j \rceil$$

ii. Calculate confidence score of the *Evaluator Model* for the i-th question:

$$Confidence^{(i)} = 1 - \sum_{k=1}^{l} \frac{|\lfloor r_k \rceil + r_k|}{l}$$

f. If $\lambda > 0.5$ and $l > 1$:

i. Calculate midpoint of the $K^{(i)}$ and evaluate both halves separately:

$m = \lfloor l/2 \rfloor, K_1 = K^{(i)}[0{:}\,m-1], K_2 = K^{(i)}[m{:}\,l]$

$$evaluate(c^{(i)}, K_1, n) \rightarrow (r_1, Maj_1, Confidence_1)$$

$$evaluate(c^{(i)}, K_2, n) \rightarrow (r_2, Maj_2, Confidence_2)$$

ii. Combine the results:

$$r^{(i)} = concat(Maj_1, Maj_2)$$

$$Maj^{(i)} = concat(Maj_1, Maj_2)$$

$$Confidence^{(i)} = \frac{Confidence_1 + Confidence_2}{2}$$

Question Type → ↓ Model	Primary Working Diagnosis (n=340)	Extracted Symptoms (n=340)	Extracted Risk Factors (n=340)	Immidiate Diagnostics Procedures or Test (n=340)	Therapeutic Strategies to Manage the Disease (n=340)	Differential Diagnoses (n=340)	Possible Complications and Management (n=306)	Long-term Management, Surveillance and Follow-up (n=204)	Treatment Strategies (n=51)
claude-3-haiku-20240307	10.0%	1.3%	3.3%	20.9%	18.1%	53.5%	30.6%	19.2%	38.9%
claude-3-opus-20240229	5.0%	2.5%	2.7%	17.2%	12.4%	55.7%	20.4%	15.6%	47.2%
dbrx-instruct	10.0%	0.5%	1.5%	21.6%	14.7%	48.6%	33.7%	28.2%	77.8%
gemma-7b-it	25.0%	3.5%	18.8%	55.2%	53.1%	**82.3%**	65.4%	50.9%	**97.2%**
gpt-3.5-turbo-1106	0.0%	3.1%	11.5%	25.6%	24.3%	48.8%	32.4%	23.9%	25.0%
gpt-4-1106-preview	5.0%	5.8%	3.3%	12.4%	12.1%	43.0%	22.5%	12.6%	30.6%
gpt-4-turbo-2024-04-09	5.0%	1.1%	4.0%	13.9%	12.7%	48.1%	24.6%	11.2%	25.0%
Llama-2-70b-chat-hf	25.0%	1.5%	10.6%	18.8%	18.0%	59.0%	24.4%	11.4%	50.0%
Llama-2-7b-chat-hf	30.0%	0.8%	11.7%	23.7%	22.4%	61.0%	33.4%	20.8%	86.1%
Llama-3-70b-chat-hf	10.0%	3.4%	7.5%	20.9%	12.8%	43.5%	22.1%	19.6%	36.1%
Llama-3-8b-chat-hf	10.0%	3.6%	19.7%	19.4%	20.6%	54.0%	27.0%	23.1%	58.3%
meditron-7b-chat	**35.0%**	**25.3%**	**47.0%**	**66.7%**	**56.6%**	81.9%	**73.2%**	**64.9%**	66.7%
medllama2_7b	15.0%	9.2%	34.6%	31.7%	28.4%	75.1%	44.7%	27.4%	58.3%
Mistral-7B-Instruct-v0.2	25.0%	3.4%	11.5%	20.4%	17.9%	60.4%	22.5%	19.3%	58.3%
Mixtral-8x22B-Instruct-v0.1	10.0%	0.0%	4.8%	19.2%	16.6%	60.4%	31.6%	20.5%	38.9%
Mixtral-8x7B-Instruct-v0.1	20.0%	3.0%	6.0%	18.0%	15.2%	47.1%	22.1%	17.2%	19.4%
WizardLM-2-8x22B	15.0%	1.0%	3.3%	12.7%	12.1%	45.6%	24.3%	16.3%	41.7%

Supplementary Figure 1: Model deficiency scores vs. question type. This figure presents performance deficiencies percentages for various AI models across different clinical question types. Models are listed vertically, while question types are arranged horizontally. GPT-3.5

and GPT-4 models consistently show low deficiency scores across most categories. Models like medilama2_7b and gemma-7b-it exhibit higher deficiencies overall. "Treatment Strategies", "Differential Diagnoses" and "Possible Complications and Management" are challenging for most models. Models generally perform well on "Extracted Symptoms" and "Extracted Risk Factors". "Primary Working Diagnosis" and "Treatment Strategies" show high variability in performance across models.

↓ Case / Question Type →	Primary Working Diagnosis (n=340)	Extracted Symptoms (n=340)	Extracted Risk Factors (n=340)	Immidiate Diagnostics Procedures or Test (n=340)	Therapeutic Strategies to Manage the Disease (n=340)	Differential Diagnoses (n=340)	Possible Complications and Management (n=306)	Long-term Management, Surveillance and Follow-up (n=204)	Treatment Strategies (n=51)
Breast cancer	11.8%	5.9%	19.1%	31.1%	19.1%	38.2%		36.5%	**58.8%**
Lung cancer	29.4%	6.9%	10.3%	28.2%	40.1%	85.3%	50.0%	**65.4%**	
Prostate cancer	**64.7%**	3.9%	2.0%	23.0%	26.9%	59.8%	23.1%	19.6%	43.6%
Colon carcinoma	5.9%	0.0%	1.5%	22.6%	1.5%	22.1%		49.5%	48.5%
Kidney cancer	29.4%	0.0%	5.9%	26.2%	21.5%	67.8%	22.7%		
Hypertension	29.4%	0.0%	16.9%	**44.4%**	11.0%	43.9%	23.5%	19.6%	
Ischemic heart disease	5.9%	6.9%	13.7%	30.9%	7.6%	34.1%	14.6%	17.0%	
Acute chest pain / myocardial infarction	35.3%	2.9%	9.4%	22.0%	41.5%	59.8%	19.8%		
Heart failure	23.5%	11.2%	7.4%	23.8%	**46.8%**	78.2%	52.2%		
Anaphylaxis	5.9%	1.2%	5.9%	15.3%	32.1%	66.2%	44.0%		
Asthma exacerbation	0.0%	2.9%	18.6%	18.6%	38.1%	41.2%	35.8%	7.4%	
Chronic obstructive pulmonary disease (COPD)	0.0%	2.9%	4.4%	17.6%	15.3%	62.4%	22.7%		
Liver cirrhosis	0.0%	0.0%	18.8%	9.3%	33.3%	**91.8%**	35.6%		
Acute kidney injury	29.4%	4.9%	2.9%	32.8%	7.8%	76.5%	13.2%	16.2%	
Chronic kidney disease	0.0%	0.0%	12.7%	17.1%	13.2%	54.4%	32.8%	12.8%	
Type 2 Diabetes Mellitus	5.9%	0.0%	16.8%	44.3%	8.8%	64.0%	30.3%		
Acute appendicitis	0.0%	0.0%	11.8%	20.6%	27.0%	74.3%	56.9%		
Stroke	0.0%	11.8%	11.8%	23.9%	11.5%	40.0%	**61.4%**	3.5%	
HIV/AIDS	23.5%	2.2%	**42.6%**	14.4%	11.8%	55.3%	19.4%	8.2%	
Major depressive disorder	0.0%	**17.6%**	4.9%	25.9%	17.9%	23.5%	29.4%	28.2%	

Supplementary Figure 2: Case deficiency scores vs. question type. This figure displays the performance deficiency scores for various clinical cases across different question types in the AMEGA benchmark. Cases are listed vertically on the left, while question types are arranged horizontally across the top. The percentages indicate the degree of deficiency for each case-question combination. This visualization allows for quick identification of which clinical cases present the greatest challenges across different aspects of medical reasoning and decision-making, highlighting areas where AI models consistently struggle or excel.

Supplementary Data 2: Template for adding cases

Clinical Content: This section will focus on the medical details of the case, including:

- Case description: A comprehensive description of the patient's presentation, medical history, and relevant findings.
- Questions: A series of open-ended questions designed to assess the LLM's clinical reasoning and guideline adherence.
- Sections/Reask questions: A breakdown of each question into more specific sub-tasks, with optional "reask" prompts to allow the LLM to refine its answers.
- Evaluation criteria/scoring system: Precise criteria and a scoring rubric for evaluating the LLM's responses based on established medical guidelines.

Technical Structure:

The technical structure of the benchmark follows a tree format, with each case being broken down into various levels for automated evaluation. The tree consists of four main levels:

1. Case description: This is the top-level description of the clinical case. It is stored separately and identified by a unique case_id. The description provides the context for the questions that follow.
2. Questions: Each case contains several questions related to the clinical scenario. These questions probe different aspects of clinical reasoning, such as diagnosis, treatment, or patient management. Each question is identified by a unique question_id and is associated with the corresponding case_id.
3. Sections and reask questions: Within each question, there are sections that break down the problem into more specific tasks. These sections can contain reask questions if the initial response does not meet the criteria fully. Every section is assigned a section_id, and like questions, is linked to the appropriate question_id and case_id.
4. Evaluation criteria and scores: At the lowest level, each section has a set of predefined evaluation criteria that dictate how the LLM's response will be assessed. Each criterion includes conditions that must be met for the response to be deemed correct. These criteria are assigned unique criterion_ids and are linked to their corresponding section_id, question_id, and case_id. The scoring system, which assigns points to the response that meets the criteria, is also defined here.

Each level of this tree is stored in separate CSV files, with relational mappings between them

using the IDs (case_id, question_id, section_id, criterion_id). This design allows for efficient automated evaluation, functioning similarly to a relational database where each evaluation criterion can be matched against the appropriate case, question, and section.

By following this template, users can seamlessly integrate new clinical cases into the benchmark. The technical structure ensures that the addition of new cases, questions, and criteria can be easily automated, while maintaining the integrity of the evaluation process.

Supplementary Data 3: Example Case Scenario

Case Scenario:

A 45-year-old woman presents with fatigue, weight gain, and cold intolerance. She reports feeling sluggish and notes that her skin is dry. Laboratory tests reveal elevated thyroid-stimulating hormone (TSH) levels.

Question:

Based on the clinical presentation and laboratory findings, what is the most likely diagnosis? Please provide the key clinical features that support your diagnosis.

Initial Model Response:

The most likely diagnosis is hypothyroidism.

Evaluation:

The model correctly identifies the diagnosis but does not provide the supporting clinical features as requested.

Reask Prompt:

Please review your answer and include the key clinical features from the case that support your diagnosis.

Revised Model Response:

The most likely diagnosis is hypothyroidism. This is supported by the patient's fatigue, weight gain, cold intolerance, sluggishness, dry skin, and elevated TSH levels.

Explanation:

In this example, the model's initial response is incomplete but not critically flawed. The 'Reask' process is applied to prompt the model to provide a more thorough answer, mirroring how a clinician might be asked to elaborate during a consultation or examination.

Example Where 'Reask' Is Not Applied

Case Scenario:

A 60-year-old man presents to the emergency department with sudden onset of severe chest pain radiating to his left arm, shortness of breath, and sweating. He appears anxious and pale. An electrocardiogram (ECG) shows ST-segment elevation in leads II, III, and aVF.

Question:

What is the immediate management for this patient?

Initial Model Response:

The patient should be scheduled for an outpatient stress test to evaluate his chest pain.

Evaluation:

The model fails to recognize the signs of an acute inferior myocardial infarction (heart attack) and provides an inappropriate management plan that delays critical treatment.

Reask:

Not applied.

Explanation:

In this critical scenario, failing to identify and appropriately manage an acute myocardial infarction could result in severe harm or death. In clinical practice, such an oversight is unacceptable due to the urgency of the situation. Therefore, the 'Reask' process is not applied, and the initial incorrect response is considered final to reflect the importance of immediate and accurate clinical decision-making in life-threatening conditions.

Supplementary Data 4: Criteria for evaluating responses

Evaluation criteria for true or false answers

The determination of whether a candidate's answer is True or False is based on its alignment with the ground truth diagnosis and the specificity and definitiveness of the response. The following criteria guide this evaluation:

Criteria for evaluating as True:

1. Direct match:
   - The answer explicitly states the ground truth diagnosis without ambiguity or alternatives.
   - *Example*: If the ground truth is "Prostate Cancer", an answer stating "Prostate cancer" is marked as True.
2. Specific subtypes or variants:
   - The answer specifies a subtype or variant within the broader category of the ground truth diagnosis.
   - *Example*: If the ground truth is "Invasive Breast Cancer", an answer stating "Invasive ductal carcinoma (IDC)" is considered True, as IDC is a subtype of invasive breast cancer.
3. Consistent terminology:
   - The answer uses terminology consistent with the ground truth diagnosis, including additional relevant details such as severity, stage, or progression.
   - *Example*: If the ground truth is "Chronic Kidney Disease", an answer stating "Chronic Kidney Disease (CKD) stage 3" is marked as True because it provides consistent, detailed information.
4. Diagnosis with relevant cause:
   - The answer identifies the diagnosis and links it to a known, relevant cause.
   - *Example*: If the ground truth is "Acute Myocardial Infarction", an answer stating "Acute Myocardial Infarction (AMI) due to plaque rupture" is marked as True because it accurately relates the diagnosis to a pertinent cause.

Criteria for evaluating as False:

1. Ambiguity or alternative possibilities:
   - The answer introduces ambiguity by stating the diagnosis as a possibility or lists multiple conditions, including those inconsistent with the ground truth.

   - *Example*: If the ground truth is "Prostate Cancer", an answer stating "Benign Prostatic Hyperplasia (BPH) with possible prostate cancer" is marked as False due to the uncertainty introduced.
2. Different condition or diagnosis:
   - The answer specifies a condition or diagnosis that does not match or directly relate to the ground truth.
   - *Example*: If the ground truth is "Acute Kidney Injury", an answer stating "Dehydration" is marked as False because dehydration is a potential cause, not the diagnosis itself.
3. General terminology without specificity:
   - The answer uses general terms lacking sufficient specificity required by the ground truth diagnosis.
   - *Example*: If the ground truth is "Invasive Breast Cancer", an answer stating "Breast cyst" is marked as False because it refers to a different, non-cancerous condition.
4. Non-specific references:
   - The answer refers to symptoms, risk factors, or non-specific conditions without explicitly confirming the diagnosis.
   - *Example*: If the ground truth is "Liver Cirrhosis", an answer stating "Chronic liver disease" is marked as False because it does not specifically confirm cirrhosis.

By adhering to these criteria, evaluations remain consistent and objective, ensuring that only answers clearly and directly aligning with the ground truth are marked as True, while those introducing ambiguity or unrelated information are marked as False.

Results of the evaluation analysis

Evaluation outcomes

The table below summarizes the comparison between the evaluator's predictions and human evaluations:

		Human Evaluation		
		**True**	**False**	**Sum**
**Evaluator Prediction**	**True**	285	5	290
	**False**	8	42	50
	**Sum**	293	47	340

Statistical metrics:

Precision: 0.98 ± 0.04; Recall: 0.97 ± 0.05; Accuracy: 0.96 ± 0.05; F1: 0.97 ± 0.05

The high F1 score (>95%) indicates strong alignment between GPT-4's evaluations and human judgments. The higher number of False Negatives compared to False Positives indicates the tendency of GPT-4 to be very cautious in its assessments.

Analysis of Delta values

The delta represents the change in the proportion of correct answers before and after human evaluation:

Correct answers before human evaluation:

$$Proportion = \frac{True\ Positives + False\ Positives}{Total\ Samples} = \frac{285 + 5}{340} = 0.85 \pm 0.10$$

Correct answers after human evaluation:

$$Proportion = \frac{True\ Positives + False\ Negatives}{Total\ Samples} = \frac{285 + 8}{340} = 0.86 \pm 0.10$$

Delta:

$$\Delta = After\ Human\ Evaluation - Before\ Human\ Evaluation = 0.86 - 0.85$$
$$= 0.01 \pm 0.04$$

<u>Interpretation:</u>

Positive Delta: Indicates the evaluator underestimated the generator's performance compared to human evaluation.

Negative Delta: Indicates the evaluator overestimated the generator's performance compared to human evaluation.

In this analysis, the slight positive delta suggests a minimal underestimation by the evaluator, which is within the expected variability range.

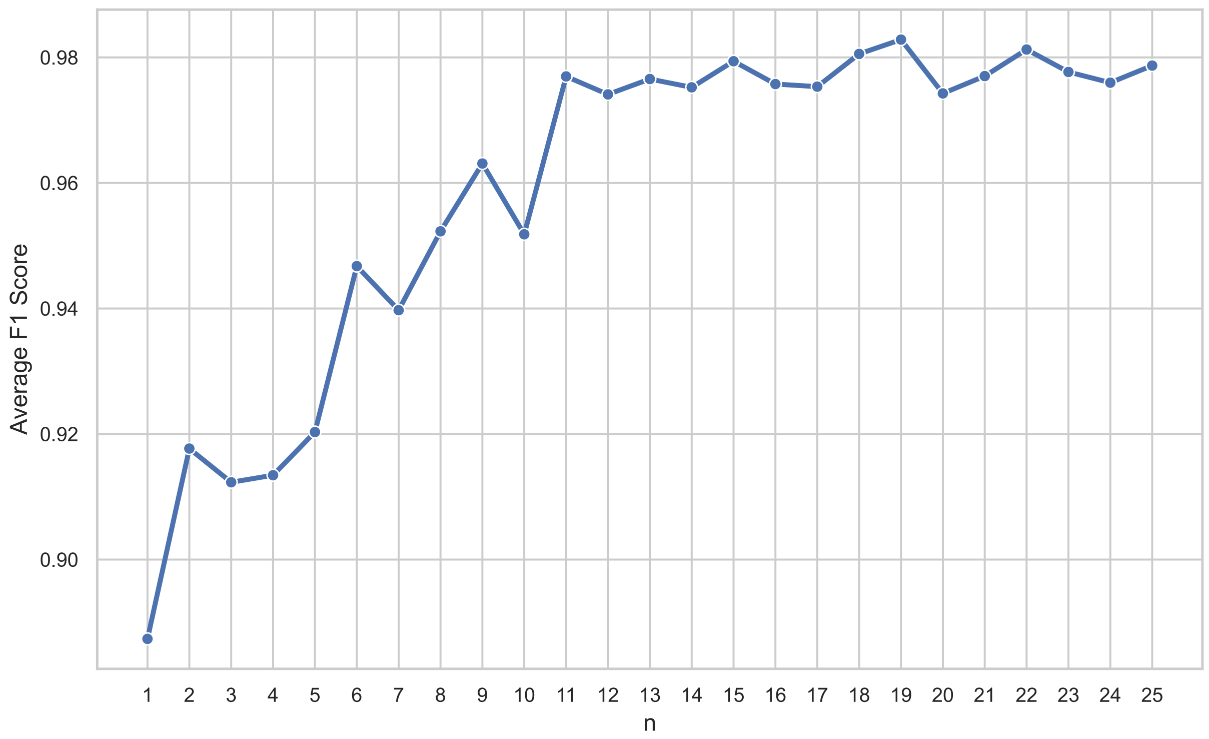

Supplementary Figure 3. Average F1 score for different amount of evaluation attempts (n) for evaluation of answers by GPT-4 and Llama-3-70B, pooled across Cases 3 and 5. F1 scores were calculated by comparing the majority vote for particular amount of evaluation attempts to the validation results by a human expert and then averaged across all 4 runs. The F1 score remains relatively constant after n = 11. Therefore, only 25 runs are depicted in the current figure.

Supplementary Data 5: Criteria for evaluating responses

Detailed case example

To illustrate how the AMEGA benchmark is applied, we provide a complete example using one of the cases from our dataset. This detailed case study demonstrates the question-and-answer process, the model's responses, and the scoring for each section.

Case ID: 1

Case Specialty: Oncology / Gynecology

Case Title: Management of breast cancer

Case Description: A 58-year-old female visits the outpatient clinic with concerns about a persistent lump in her left breast. She first noticed the lump and dimpling of the surrounding skin while showering about 8 weeks ago. Over the past month, she has also observed a subtle retraction of the nipple with redness in the nipple area and blood-tinged discharge, but no pain, as well as a small lump in her left armpit. The patient has never given birth. She admits to skipping her scheduled mammogram for the past two years due to anxiety. Upon further history, she reveals that she went through menopause at age 51 and has been on hormone replacement therapy for the past 5 years to manage her post-menopausal symptoms. She has not experienced any weight loss, fatigue or abdominal or bone pain. Her sister was diagnosed with breast cancer at age 31, and her paternal grandmother had a history of uterine cancer. She has never smoked or consumed alcohol excessively.

Question ID	Question Text	Section Reask Text	Evaluation Criterion	Score
1	Based on the patient's presentation and past medical history, what is your primary working diagnosis? Please justify your response using the provided clinical information.	Reask is NOT allowed	Invasive Breast Cancer as the primary working diagnosis	5
		Which are further symptoms of invasive breast cancer, obtainable	Lump/mass in the breast	0.5
			Redness in the nipple area	0.5

		from the above case report?	Retraction of the nipple	0.5
			Blood-tinged discharge	0.5
			Dimpling of the skin	0.5
			Lump or swelling in the armpit/underarm lymph nodes	0.5
2	What are significant risk factors for your primary working diagnosis obtainable from the above case report?	Which are further risk factors for invasive breast cancer obtainable from the above case report?	Nulliparity (not given birth) and/or absence of breastfeeding	0.5
			Early menarche and/or late menopause	0.5
			Hormone replacement therapy after menopause	0.5
			Acknowledgment of the patient's family history as a genetic predisposition (first-degree relatives such as sister with breast cancer)	0.5
3	Detail all the immediate diagnostic procedures or tests you would perform to confirm your diagnosis. Provide details about how each test should be performed and why.	Which procedure should be performed first in patients with suspected breast cancer?	Mention the need of a diagnostic mammogram	1
			Mammogram should be specified as a bilateral mammogram	1
			Each mammogram should contain two low-dose x-rays of the breast, one in	0.5

			cranio caudal view and one in mediolateral oblique	
		Which other imaging examinations should also be considered in patients with invasive breast cancer?	Ultrasound as necessary (e.g., also for assessment of axillary nodes)	0.5
			Breast ultrasound if mammography is inconclusive	0.5
			Optional MRI, with special consideration for mammographically occult tumors	0.5
			Breast MRI scans being conducted using IV contrast and executed and analyzed by a skilled breast imaging team	0.5
			Additional imaging studies, such as MR and specifically CT staging, as clinically indicated	0.5
			CT scan of the chest, with or without contrast	0.5
			CT scan with contrast of the abdomen and possibly pelvis, or MRI with contrast	0.5
			Bone imaging via a bone	0.5

			scan or sodium fluoride PET/CT	
		What procedures should be performed to determine pathology in patients with invasive breast cancer?	The answer should include the performance of a core needle biopsy	0.5
			Should mention clip placement	0.5
		Which procedure should be performed in patients with invasive breast cancer if core needle biopsy is not feasible?	Surgical biopsy if core needle biopsy is not feasible	0.5
			Surgical biopsy as an alternative in case of inconclusive results from core needle biopsy	0.5
		Patients with newly diagnosed breast cancer should undergo biopsy. Why is this recommended, what is tested and what should one do, if an initial biopsy sample yields inconclusive results?	HER2 testing, following the procedures specified in the ASCO/CAP HER2 testing guideline	1
			ER testing determines if a patient is suitable for endocrine therapies. Cancers are considered ER-positive when 1% to 100% of their cells show positive ER expression	1
			Retesting, if the sample was suboptimal	0.5
			Retesting, if a testing error	0.5

			is suspected	
			Retesting, o if additional samples present a higher-grade cancer distinct from the initial biopsy	0.5
			Retesting to address potential heterogeneity in a high-grade cancer	0.5
			Retesting if such retesting can inform clinical decision-making	0.5
		Which blood tests should be performed in patients with breast cancer?	Metabolic panel	1
		Which additional testing and assessment could be indicated in patients with invasive breast cancer, especially young patients?	Genetic testing recommended (BRCA1, BRCA2)	1
			Genetic testing recommended, if patient susceptible to inherited breast cancer	1
4	Assume the diagnosis of an invasive breast cancer is confirmed with a clinical stage cT2, cN+, M0. The patient is a BRCA2 carrier, and ER-positive and HER2-negative. What are the immediate,	Could you explain the recommended diagnostic and treatment approaches for a breast cancer patient who is a candidate for chemotherapy, has ER-positive and HER2-	21-gene RTPCR assay	1
			Supplementary Olaparib considering the BRCA2 mutation	1
			Adjuvant chemotherapy	1

	therapeutic strategies to manage the disease? For each therapy, explain how and when it should be performed and explain alternative strategies, when the first line therapy is not indicated.	negative status with a BRCA2 mutation, and possesses a genetic predisposition to breast cancer	Endocrine therapy	1
			Total mastectomy	1
			Surgical axillary staging and with or without breast reconstruction	1
		Assuming the patient has no genetic predisposition and a lower tumor stage, which kind of surgical therapy would be appropriate in this patient?	Breast-conserving surgery (BCS)	1
			Followed by radiotherapy (after BCS and axillary staging)	1
5	During surgical axillary staging, the patient has 4 positive axillary nodes. Surgical margins are negative. What treatment should be performed?	Which non-surgical treatment is recommended in this patient?	Whole breast radiation therapy	1
			Combined with radiation therapy to chest wall and extensive regional nodal irradiation	1
6	What management is recommended for surveillance and follow-up in a patient with invasive breast cancer according to current clinical guidelines?	What further management is recommended for surveillance and follow-up in a patient with invasive breast cancer according to current NCCN guidelines?	History and physical exam 1-4 times yearly for 5 years, then once a year	1
			Periodically review family history for changes and refer for genetic counselling when needed	1
			Offer guidance on lymphedema management	1

			Schedule mammograms every 12 months; no routine imaging needed for reconstructed breasts. For those with germline mutations or a family history of breast cancer, refer to specific guidelines	1
			No laboratory or imaging studies needed unless there are signs or symptoms of recurrent disease	1
			Monitor for cardiotoxicity in patients who had specific treatments. Offer guidance on the risk of other health conditions	1
			Emphasize adherence to adjuvant endocrine therapy. For patients on tamoxifen, conduct age-appropriate gynecologic screening. Monitor bone health in patients on specific treatments	1
			Encourage an active lifestyle, healthy diet, limited alcohol intake, and maintaining a BMI of 20-25 for best outcomes.	1

			Promote care coordination between primary and specialty providers. Provide a personalized survivorship treatment plan	1
			Regularly encourage patients to ensure they adhere to screenings and medications	1
7	Given that the primary diagnosis is breast cancer, list the main differential diagnoses that you should also consider for a patient presenting with a lump in the breast. Discuss how you would differentiate these from breast cancer based on clinical presentation and investigations.	What are other common differential diagnoses for breast cancer?	Breast abscess: Associated with pain and inflammation	1
			Fat necrosis: Often associated with a history of trauma, surgery, or radiation to the breast	1
			Fibroadenoma: Often presents in younger women in their 20s and 30s; usually appears well circumscribed on imaging	1
			Intraductal papilloma: While intraductal papilloma often presents with nipple discharge, it typically does not present as a palpable lump, shows well-defined margins on imaging.	1
